# Clinical significance of field safety notices concerning implantable pacemakers and defibrillators

**DOI:** 10.1093/europace/euag031

**Published:** 2026-02-23

**Authors:** Yijun Ren, Alessandra Baruffini, Sofia Biolo, Rita Corti, Jens Cosedis Nielsen, Alan G Fraser, Enrico G Caiani

**Affiliations:** Department of Electronics, Information and Bioengineering, Politecnico di Milano, P.zza L. da Vinci 32, Milan 20123, Italy; Department of Electronics, Information and Bioengineering, Politecnico di Milano, P.zza L. da Vinci 32, Milan 20123, Italy; Department of Electronics, Information and Bioengineering, Politecnico di Milano, P.zza L. da Vinci 32, Milan 20123, Italy; Department of Electronics, Information and Bioengineering, Politecnico di Milano, P.zza L. da Vinci 32, Milan 20123, Italy; Department of Cardiology, Aarhus University Hospital and Department of Clinical Medicine, Aarhus University, Aarhus, Denmark; Department of Cardiology, University Hospital of Wales, Heath Park, Cardiff CF14 4XW, UK; Department of Electronics, Information and Bioengineering, Politecnico di Milano, P.zza L. da Vinci 32, Milan 20123, Italy; IRCCS Istituto Auxologico Italiano, Ospedale S. Luca, Milan 20149, Italy

Pacemakers (PPM) and implantable defibrillators (ICD) need to be safe and effective, with excellent long-term performance. The European Union (EU) Medical Device Regulation (MDR) 2017/745 that entered into force in May 2021^1^ introduced more rigorous post-market surveillance (PMS). Every manufacturer must establish a PMS system to collect data on the safety and performance of its devices actively and systematically throughout their lifecycle. PPM and ICD are susceptible to hardware, software, and battery malfunctions that sometimes result in large-scale recalls. For example, in 2021 the US Food and Drug Administration (FDA) issued Class I recalls (the most severe category) for certain St. Jude (Abbott) and Boston Scientific pacemakers due to premature battery depletion, which affected 97 000 and 72 000 units, respectively.

Whenever a problem is identified, the manufacturer must notify its national competent authority (CA) (regulatory agency) and issue a field safety notice (FSN) in order to prevent or minimize the risk of more events. The CA may also issue a device alert to advise doctors and patients. The level of detail, completeness, and utility of FSNs can vary due to local regulatory requirements, inconsistent reporting practices, and/or the lack of a standard reporting format.^2^ The MDR was intended to improve transparency and coordination through the EUropean DAtabase on MEdical Devices (EUDAMED), but its clinical module has been delayed.

Currently, FSNs can be retrieved only by separate queries to national CA websites. Duty to inform stakeholders has led to the issuance of multiple FSNs, and it can be challenging for clinicians to interpret these real-world data. Therefore, to identify the most frequent issues and advise on their significance, we developed a PMS tool capable to retrieve all historical FSNs from multiple EU and non-EU national CA official websites.^3,4^ The study was undertaken within the EU-funded CORE-MD project (Coordinating Research and Evidence for Medical Devices) which was led by the European Society of Cardiology.^5^

Automated web-scraping was employed to aggregate reports from 14 EU countries (Croatia, Czechia, Denmark, Estonia, France, Germany, Ireland, Italy, Latvia, Poland, Slovenia, Spain, Sweden, the Netherlands) and from 6 countries outside the EU (Australia, Brazil, Canada, Switzerland, the UK, and the USA). We identified 625 FSNs, published between 1 January 2014 and 31 December 2024, and categorized under European Medical Device Nomenclature codes J0101 (implantable pacemakers) or J0105 (implantable defibrillators).,Additional 331 FSNs were found by querying the PMS tool database using manufacturers’ and devices’ names. After duplicates removal, 121 FSNs were analysed: full reports were retrieved, and relevant device problems were classified by four observers according to International Medical Device Regulators Forum (IMDRF) codes^6^; at IMDRF Level 1, 86% were coded consistently by all raters. As one FSN may report multiple findings, the final total was 124 FSNs matched to their problem categories.

The largest proportion of all reports came from the USA (23% of 956), followed by Italy (7%), while of unique reports from Medtronic (32% of 124), Boston Scientific (31%), or Abbott (26%). The commonest problems were coded as ‘electrical or electronic’ (IMDRF code A07; 26% of 124) and ‘manufacturing, packaging’ (A02; 11%); at a more detailed level, the most frequent were ‘premature discharge of battery’ (A070504; 9%), ‘non-standard device’ (A020102; 8%), and ‘battery problem: high impedance’ (A070501; 5%) (*Figure 1*).

**Figure 1:**
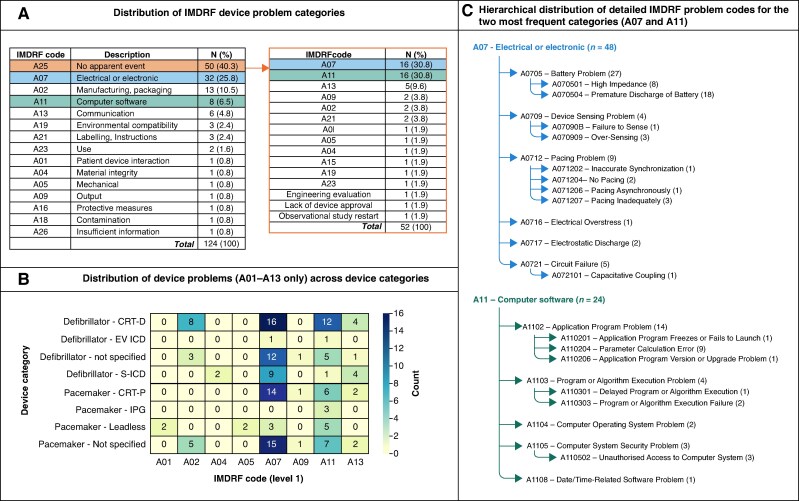
IMDRF-based categorization of device problems reported in field safety notices related to implantable pacemakers and defibrillators from 20 countries, published between 1 January 2014 and 31 December 2024. (*A*) Summary tables of reported device problems categorized using IMDRF codes, illustrating the overall code distribution (124 unique FSN–problem pairs) and the reassignment of FSNs originally classified as ‘A25—no apparent event’. Although 50 FSNs were labelled as A25, reassignment yielded 52 problem entries (one FSN could report multiple problems). (*B*) Distribution of IMDRF codes across device categories following reassignment of FSNs labelled as A25. Counts are based on 171 unique FSN–problem–device–category combinations. Only problem categories A01–A13 are shown; colour intensity indicates frequency. (*C*) Hierarchical distribution of detailed IMDRF problem codes for the two most frequently reported problem categories after reassignment of A25 FSNs (A07 and A11). Numbers in parentheses indicate the number of FSNs assigned to each code. Counts for detailed subcodes may not sum to the parent code, as some FSNs were classified only at the parent level.

The largest group of FSNs (*n* = 50; 40%) were initially classified under IMDRF code A25 ‘no apparent event’, because they were updates on earlier reports; subsequently, they were re-analysed in detail. As a result, we identified 52 underlying problems which were mostly electrical (A07; 16/52, 31%) or related to computer software (A11; also 31%). Specific issues that were reported infrequently but potentially of clinical importance included ‘failure to sense’, ‘application program freezes or fails to launch’, ‘application program version or upgrade problem’, ‘delayed program or algorithm execution’, and ‘unauthorized access to computer system’. In *figure 1*, FSNs first coded as A25 are designated according to their underlying problems (panel A).

Finally, we identified specific brand names mentioned in FSNs and grouped them into device types (*Figure 1*, panel B). Pacemaker categories were CRT-P, IPG, and leadless; defibrillator categories were CRT-D, S-ICD, and EV-ICD. A single FSN could reference more than one device category, so each combination was considered separately, giving a total of 171 FSN–problem–device–category combinations. The predominant problems are related to electrical issues and computer software, both for pacemakers and defibrillators (*Figure 1*, panel C).

In summary, and unlike previous studies that focused on specific pacemaker models or that were limited geographically, our PMS tool provided a global perspective by retrieving 121 unique FSNs from 20 countries. The effectiveness of FSNs as a key component of PMS can be compromised by under-reporting, over-reporting driven by media coverage, and delayed notifications,^7,8^ yet they remain essential. Publication of a FSN may be followed by a ‘Field Safety Corrective Action’ with guidance to minimize adverse effects, but for clinically interpretable insights more specific details should be coded and published.^9^

Our findings imply that the main technical vulnerability for implantable pacemakers and defibrillators lies in their energy management systems and in the accuracy and performance of software. The broader literature cites early battery depletion due to poor stimulation thresholds, pulse amplitude, and pulse duration as common causes of pacemaker failure. A previous ESC report on remote monitoring of cardiac implantable electronic devices highlighted cybersecurity vulnerabilities,^10^ and our dataset included reports of unauthorized access to computer systems. Some FSNs concerned practical issues such as packaging, sterilization, or labelling; while not directly impacting the intrinsic performance of devices, these may compromise their integrity, may delay clinical use, or increase the risk of procedural complications.

For clinicians, keeping track of FSNs from different manufacturers is difficult. A readily accessible and centralized overview could improve awareness and adherence to safety communications. Our study has highlighted the need for standardized and more detailed reporting, so we recommend establishing expert groups at the national level to review FSNs, interpret their clinical impact, and provide clear guidance to healthcare professionals and patients with actionable clinical recommendations. At the EU level, expert opinions on FSNs would support regulators in their decisions about market access.

## Data Availability

The data underlying this article will be shared on reasonable request to the corresponding author.
